# Short-term clinical outcomes after implantation of monofocal intraocular lens with enhanced intermediate function in eyes with epiretinal membrane

**DOI:** 10.1038/s41598-023-44839-4

**Published:** 2023-10-21

**Authors:** Ho Seok Chung, Sanghyu Nam, Joon Hyuck Jang, Ko Eun Lee, Jae Yong Kim, Hungwon Tchah, Hun Lee

**Affiliations:** 1 Department of Ophthalmology, Asan Medical Center, University of Ulsan College of Medicine, 88, Olympic-Ro 43-Gil, Songpa-Gu, Seoul, 05505 South Korea; 2 Center for Cell Therapy, Asan Medical Center, Seoul, South Korea

**Keywords:** Health care, Medical research

## Abstract

Our study evaluated the clinical outcomes after implantation of a monofocal intraocular lens (IOL) with enhanced intermediate function in eyes with epiretinal membrane (ERM). Patients with preexisting ERM who underwent cataract surgery with implantation of monofocal IOL with enhanced intermediate function were included retrospectively. According to the ERM grade and central subfield thickness (CST) obtained from preoperative optical coherence tomography, patients were divided into non-fovea-involving and fovea-involving ERM groups. At 1 month after surgery, uncorrected distance visual acuity (UDVA), corrected distance visual acuity (CDVA), uncorrected intermediate visual acuity (UIVA), uncorrected near visual acuity (UNVA), contrast sensitivity, defocus curve, and satisfaction questionnaire were evaluated. Postoperative clinical findings were compared with age-matched controls without ERM. A total of 50 patients’ eyes (28 and 22 in the non-fovea-involving and fovea-involving ERM groups, respectively) were compared with 42 control eyes. One month post-surgery, significant differences in UDVA, CDVA, and CST (corrected *P* was < 0.001, = 0.001, and < 0.001, respectively) were observed between the fovea-involving ERM and control group; however, no significant differences in UIVA and UNVA were observed between the two groups. Contrast sensitivity showed inferior results in the fovea-involving group without significance. Photic phenomena were reported less in the fovea-involving group than in the non-fovea-involving group. More than 70% of patients in both ERM groups were satisfied. Implantation of monofocal IOL with enhanced intermediate function could be a good option for patients with ERM that need intermediate vision.

## Introduction

As the demand for near work has increased, the use of presbyopia-correcting multifocal intraocular lenses (IOLs) during cataract surgery has also increased in recent years^1^. Due to accommodation loss of monofocal IOL, most patients need to use reading glasses for near tasks^2^. Javitt et al. found that only 9.8% of the studied participants who had undergone binocular monofocal IOLs surgery did not require reading glasses for near tasks, and 80.4% needed reading spectacles for more than half of the time for near vision^3^. The advent of multifocal IOLs and related technologies has changed the approach to cataract surgery over the past few years^4^.

Eyes with macular diseases that significantly reduce macular function are relatively contraindicated for multifocal IOL implantation^5^. Although eyes with epiretinal membrane (ERM) are not good candidates for multifocal IOL implantation, the question arises whether the eyes with a clinically insignificant ERM that does not affect visual acuity are candidates for multifocal IOL implantation. In a study evaluating the clinical outcome of multifocal IOL implantation in eyes with ERM, visual improvement and multifocality were achieved by vitrectomy combined with membrane peeling and multifocal IOL implantation in patients with idiopathic ERM^6^. However, in a recent retrospective study, implantation of multifocal IOL deteriorated visual quality even in patients with mild ERM without foveal involvement, and corrected visual acuity decreased as the grade of ERM increased^7^. In addition, ERM is one of the diseases for which retinal specialists have disapproved of multifocal IOL implantation, and it is difficult for surgeons to remove the ERM of eyes implanted with multifocal IOLs^8–10^.

Eyhance IOL (Tecnis ICB00, Johnson & Johnson Vision Care Inc, Jacksonville, FL, USA) is a new monofocal IOL that could improve the intermediate-distance performance of monofocal IOLs while minimizing the undesired phenomena of multifocal IOLs. This might accelerate neural adaptation and increase the range of patients who would benefit from these IOLs^11^. Eyhance IOL meets modern needs and expectations with a modified aspheric anterior surface and a continuous power profile^12^. In a recent case series, Eyhance IOL was shown to provide satisfactory intermediate-distance spectacle independence while preserving the visual quality of the single-piece monofocal IOL produced by the same manufacturer^13^.

Considering the nature of monofocal IOLs, Eyhance IOL may be a good alternative to reduce visual phenomena while improving intermediate visual acuity in patients with ERM, in whom multifocal IOL implantation is relatively contraindicated. Therefore, in the present study, we evaluated macular involvement and ERM grading by optical coherence tomography (OCT) in eyes with ERM and compared clinical outcomes after implantation of Eyhance IOL in eyes with and without ERM.

## Results

A total of 92 patients’ eyes were included in this study. Of these 92 eyes, 28 were included in group 1 (non-fovea-involving ERM group), 22 in group 2 (fovea-involving ERM group), and 42 age-matched were included in the control group. Table 1 shows the baseline demographics of patients. There were no significant differences in any parameters between the group 1 and control group. However, in group 2, CDVA and CST were significantly different from those of the control group. There were no significant adverse effect such as postoperative cystoid macular edema during the follow-up period.

**Table 1:** Baseline characteristics of patients in all the groups.

Parameters	Group 1	Group 2	Control group	*P*^*a*^	*P*^*b*^	*P*^*c*^	*P*^*d*^
Number (eyes)	28	22	42				
Age (years)	76.7 ± 7.4	75.2 ± 7.8	75.5 ± 8.0				
Sex (M:F)	9:19	7:15	21:21				
UDVA (logMAR)	0.45 ± 0.36	0.60 ± 0.47	0.40 ± 0.23	0.409	> 0.999	> 0.999	0.507
CDVA (logMAR)	0.36 ± 0.38	0.49 ± 0.50	0.22 ± 0.25	0.048	0.468	> 0.999	0.004
AXL (mm)	24.20 ± 1.39	23.83 ± 1.41	23.52 ± 1.09	0.917	> 0.999	> 0.999	> 0.999
SE (D)	− 0.51 ± 2.34	0.01 ± 1.99	− 0.11 ± 1.48	0.121	0.414	> 0.999	0.126
Corneal astigmatism (D)	0.71 ± 0.39	0.97 ± 0.62	0.67 ± 0.44	0.113	> 0.999	0.315	0.213
CST (µm)	249.33 ± 15.56	360.00 ± 41.20	248.21 ± 12.93	< 0.001	< 0.001	> 0.999	< 0.001

Group 1, patients with non-fovea-involving ERM group; Group 2, patients with fovea-involving ERM group.

*P*^a^-value with Kruskal–Wallis test among three groups.

*P*^b^-value with Mann–Whitney test between the Groups 1 and 2 after post-hoc Bonferroni correction.

*P*^c^-value with Mann–Whitney test between the Group 1 and control group after post-hoc Bonferroni correction.

*P*^d^-value with Mann–Whitney test between the Group 2 and control group after post-hoc Bonferroni correction.

*ERM* epiretinal membrane, *UDVA* uncorrected distance visual acuity, *CDVA* corrected distance visual acuity, *AXL* axial length, *SE* spherical equivalent, *CST* central subfield thickness, *D* diopter.

**Figure 1:**
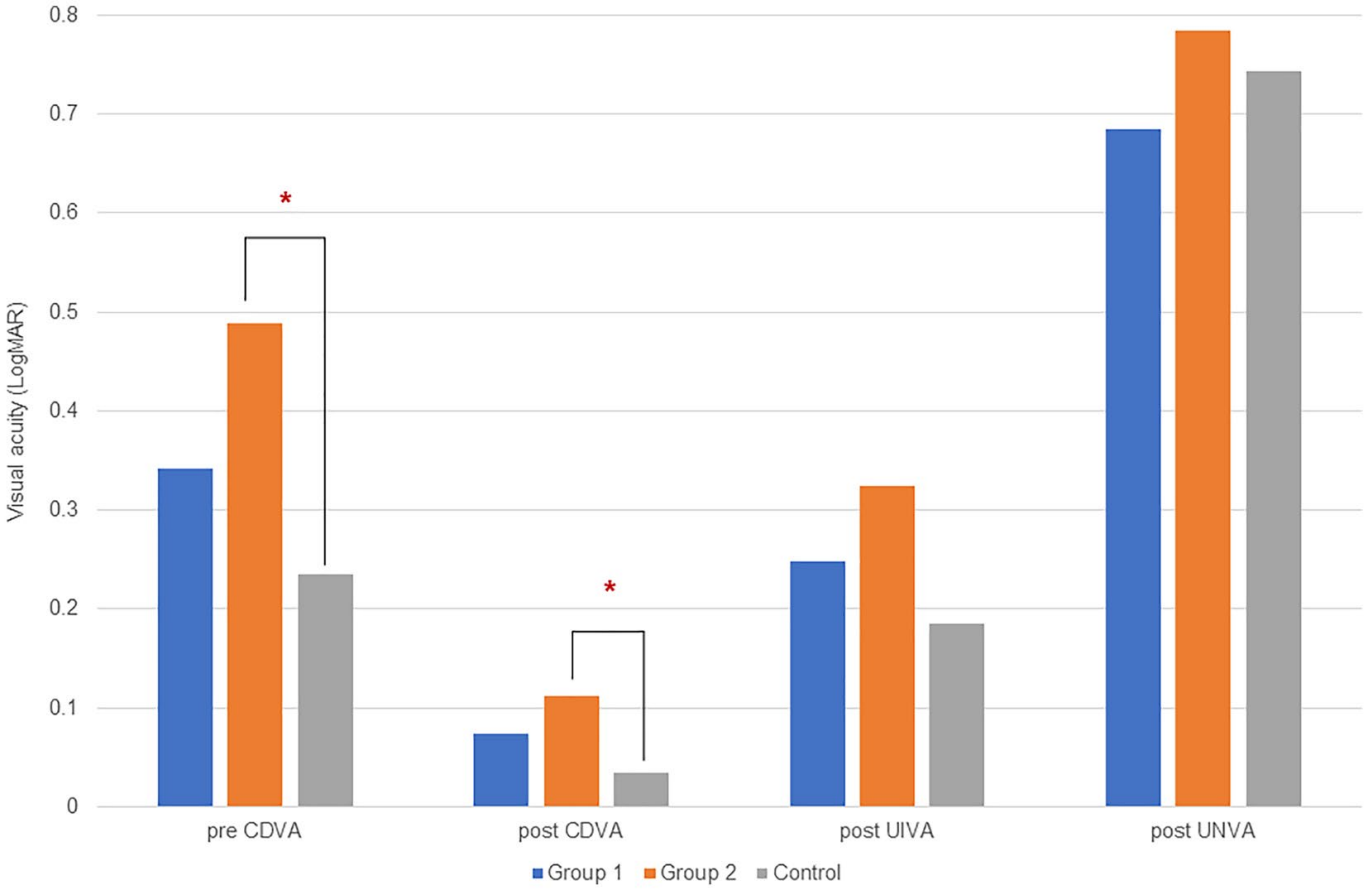
Comparison of pre-and post-operative visual acuity (LogMAR), preoperative corrected distance visual acuity (pre CDVA), postoperative corrected distance visual acuity (post CDVA), postoperative uncorrected intermediate visual acuity (post UIVA), and postoperative uncorrected near visual acuity (post UNVA) between groups. *Statistically significant difference by Kruskal–Wallis test with Bonferroni post-hoc correction.

Table 2 and Fig. 1 show the clinical findings at 1 month postoperatively. UDVA and CDVA were significantly improved after surgery in all three groups (all *P* < 0.001). There were no significant differences in UDVA, CDVA, and CST between group 1 and control group; however, significant differences in UDVA, CDVA, and CST (corrected *P* < 0.001, = 0.001, and < 0.001, respectively) were observed between the group 2 and control group. UIVA and UNVA were not significantly different among all groups. The group 1 showed a CDVA of 0.1 logMAR or better in 21 of 28 eyes (75.0%), the group 2 showed a CDVA of 0.1 logMAR or better in 16 of 22 eyes (72.7%), and all patients in the control group showed a CDVA of 0.1 logMAR or better.

**Table 2:** Clinical findings at 1 month after cataract surgery.

Parameters	Group 1	Group 2	Control group	*P*^*a*^	*P*^*b*^	*P*^*c*^	*P*^*d*^
UDVA (logMAR)	0.14 ± 0.16	0.26 ± 0.20	0.10 ± 0.11	< 0.001	0.069	0.282	< 0.001
*P*^*e*^	< 0.001	< 0.001	< 0.001				
CDVA (logMAR)	0.07 ± 0.06	0.11 ± 0.10	0.03 ± 0.05	0.001	0.600	0.102	0.001
*P*^*e*^	< 0.001	< 0.001	< 0.001				
UIVA (logMAR)	0.26 ± 0.23	0.32 ± 0.22	0.18 ± 0.13	0.071	0.969	> 0.999	0.117
UNVA (logMAR)	0.71 ± 0.19	0.78 ± 0.27	0.74 ± 0.06	0.528	0.969	> 0.999	> 0.999
SE (D)	− 0.34 ± 0.52	− 0.30 ± 0.64	− 0.32 ± 0.53	0.264	0.348	0.786	> 0.999
*P*^*e*^	0.232	0.131	0.123				
CST (µm)	255.08 ± 18.00	360.59 ± 36.52	251.87 ± 14.46	< 0.001	< 0.001	> 0.999	< 0.001
*P*^*e*^	0.201	0.463	0.241				

Group 1, patients with non-fovea-involving ERM group; Group 2, patients with fovea-involving ERM group.

*P*^a^-value with Kruskal–Wallis test among three groups.

*P*^b^-value with Mann–Whitney test between the Groups 1 and 2 after post-hoc Bonferroni correction.

*P*^c^-value with Mann–Whitney test between the Group 1 and control group after post-hoc Bonferroni correction.

*P*^d^-value with Mann–Whitney test between the Group 2 and control group after post-hoc Bonferroni correction.

*P*^e^-value with Wilcoxon signed rank test between pre- and post-operative findings.

*ERM* epiretinal membrane, *UDVA* uncorrected distance visual acuity, *CDVA* corrected distance visual acuity, *UIVA* uncorrected intermediate visual acuity, *UNVA* uncorrected near visual acuity, *SE* spherical equivalent, *CST* central subfield thickness.

**Figure 2:**
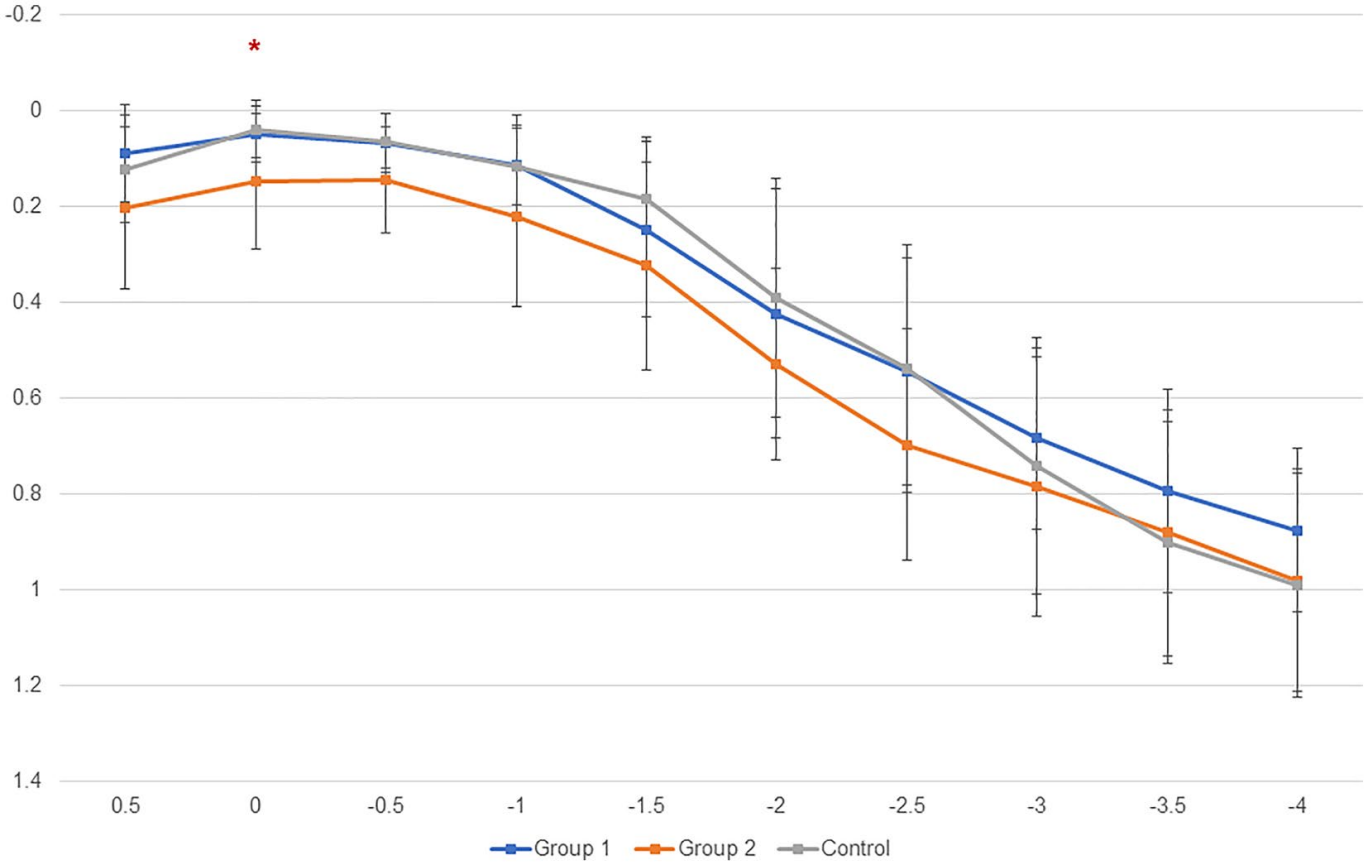
Mean monocular defocus curve of eyes implanted with Eyhance IOL. *Statistically significant difference by Kruskal–Wallis test.

Figure 2 shows the monocular defocus curve at 1 month after surgery. The group 2 showed similar visual acuity results in all areas with no significant difference from the control group. Only at a vergence of 0.00 D, the group 2 showed significantly lower visual acuity than the control group (corrected *P* = 0.005). At other vergences, the group 2 consistently showed lower visual acuity than the control group, but there was no significant difference.

**Figure 3:**
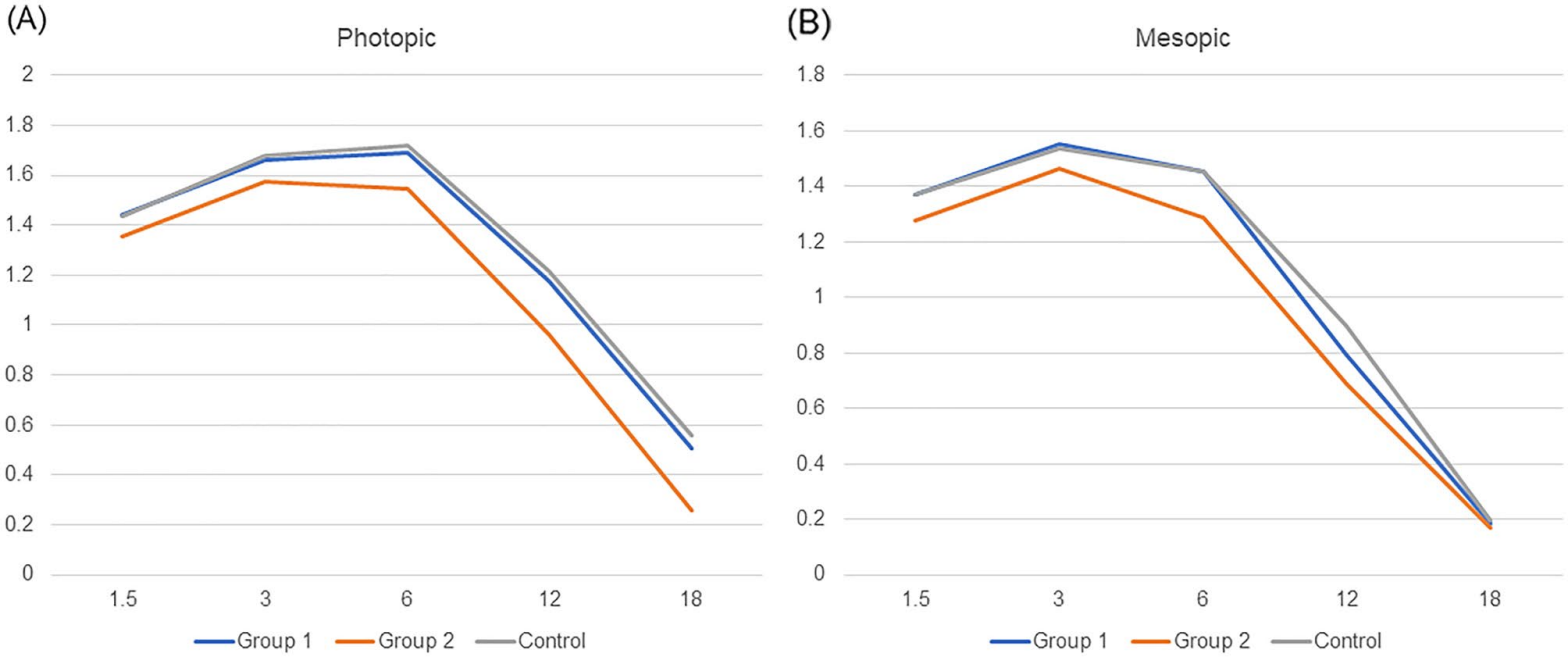
Mean monocular contrast sensitivity at 1 month after surgery. (**A**) photopic condition, (**B**) mesopic condition.

Contrast sensitivity at 1 month after surgery was measured under both photopic (Fig. 3A) and mesopic (Fig. 3B) conditions. The group 1 showed very similar results to the control group, while the group 2 showed slightly decreased contrast sensitivity; however, no significant differences were found in all spatial frequencies.

**Figure 4:**
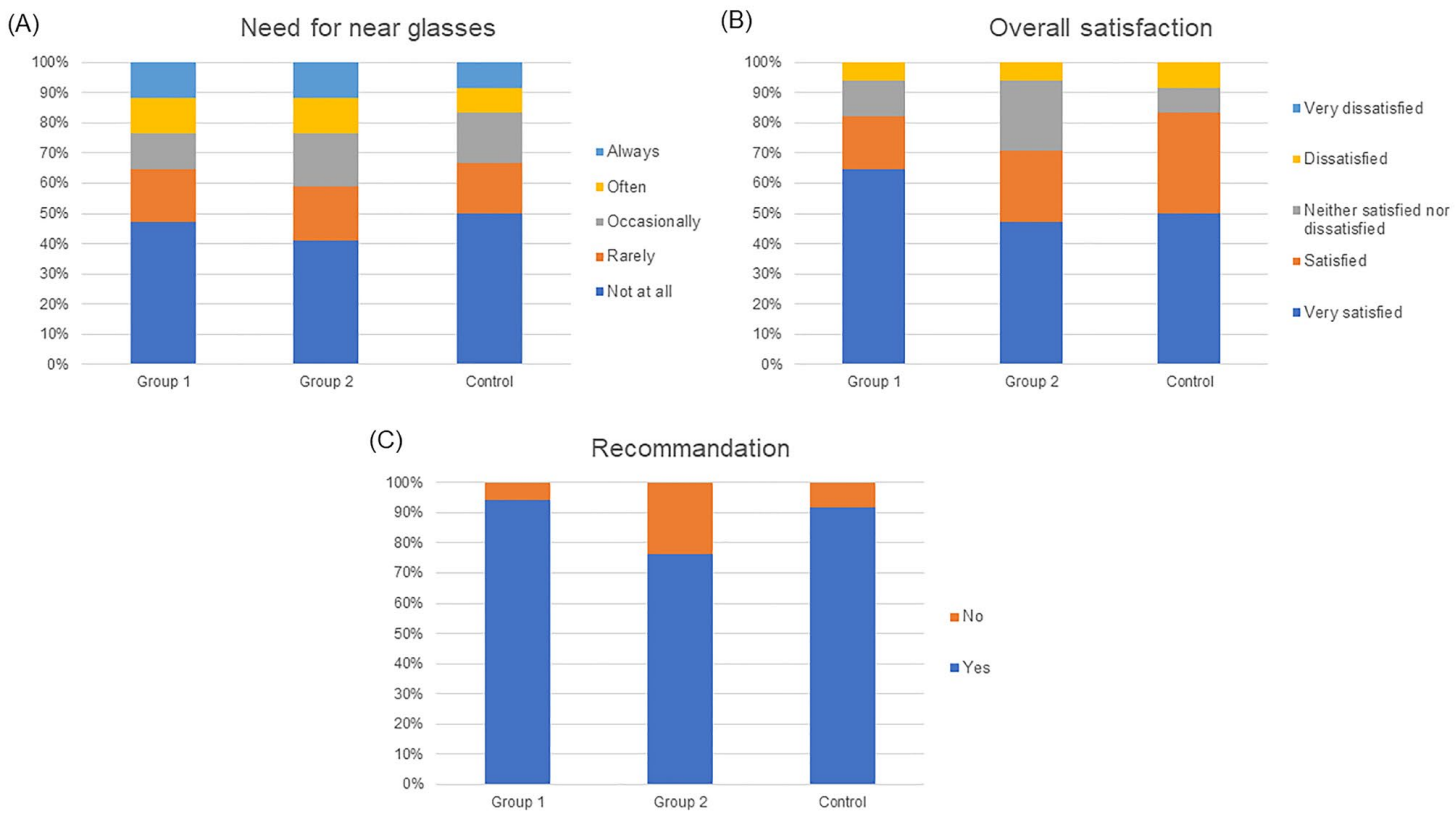
Results of subjective symptom survey using questionnaire at 1 month after surgery for (**A**) overall satisfaction, (**B**) recommendation to others, and (**C**) spectacle independence.

Figure 4 shows the subjective symptom survey results obtained from the questionnaire. In all three groups, more than half of the patients reported rarely or no need for near glasses at 1 month after surgery (Fig. 4A). Regarding overall satisfaction, the percentages of patients who answered that they were satisfied or very satisfied with their results were 82.4% in the group 1, 70.6% in the group 2, and 83.3% in the control group (Fig. 4B). In addition, 94.7% and 92.9% of the group 1 and control group, respectively, answered that they would recommend the same surgery to their family or relatives, while 76.5% of the group 2 answered that they would recommend it (Fig. 4C).

**Figure 5:**
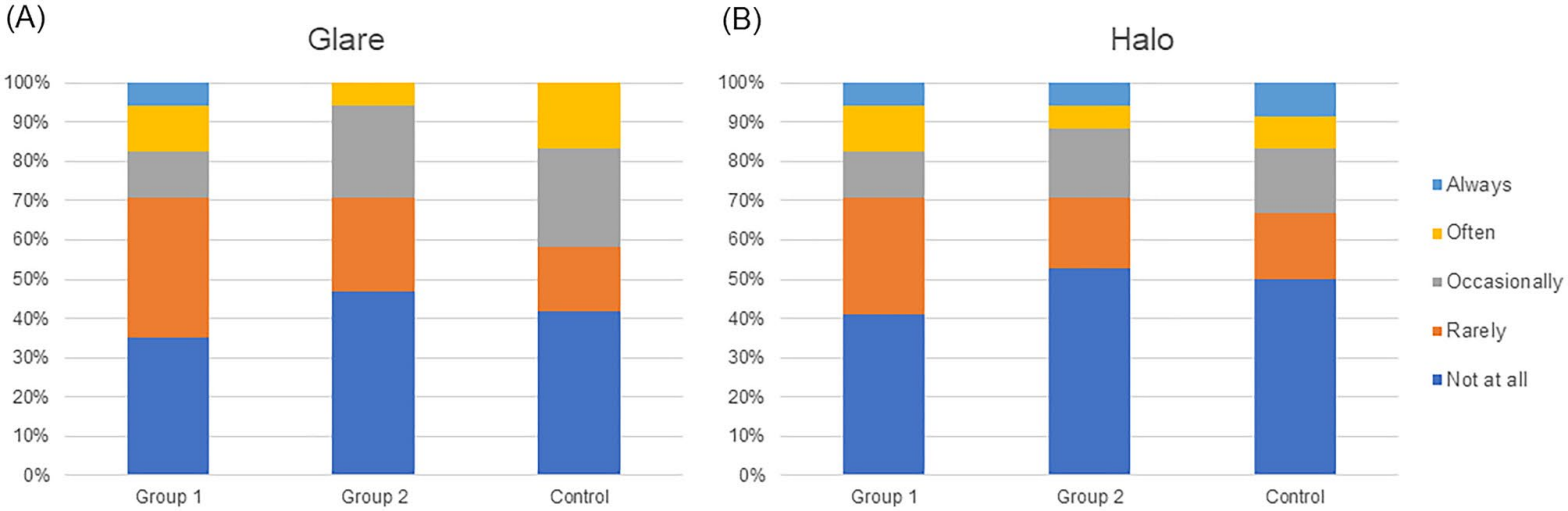
Results of subjective symptom survey using questionnaire at 1 month after surgery for (**A**) frequency of glare and (**B**) frequency of halo.

Figures 5 shows the percentage of occurrence of photic phenomena. 17.6% of the group 1 reported that they experience glare symptoms often or always, compared to 5.9% in the group 2 and 16.7% in the control group (Fig. 5A). The percentages of patients who reported often or always experiencing halo symptoms were 17.6% in the group 1, 11.8% in the group 2, and 16.7% in the control group (Fig. 5B).

## Discussion

In the present study, monofocal IOL with enhanced intermediate function (Eyhance IOL) demonstrated good subjective and objective outcomes in patients with ERM. In particular, lower visual acuity only at distant vision was observed in the group 2 than in other groups; however, there were no significant differences in UIVA, UNVA, and contrast sensitivity.

Eyhance IOL had an advantage in showing less photic phenomenon and better intermediate visual acuity than monofocal IOL in previous studies^14–16^. In patients with retinal disease, multifocal IOL implantation can bring deteriorated visual function with decreased contrast sensitivity^7^. There have been reports that the light-splitting nature of multifocal IOLs can reduce contrast sensitivity, especially in low-mesopic environments^17–19^. In addition, it has been reported that implantation of multifocal IOLs may cause disturbance of visualization during subsequent ERM removal surgery^9,10^. Although there have been no human clinical reports on macular visualization during vitrectomy surgery, the Eyhance IOL showed better macular visibility in the Gullstrand eye model, which can be considered an advantage over the multifocal IOL^20^.

In terms of postoperative visual acuity results, UDVA and CDVA were significantly improved in all groups compared to preoperative UDVA, CDVA, suggesting patients with moderate lens opacities were included in this study. Comparing the three groups, group 2 showed a significant decrease in CDVA compared to the control group, which is thought to be due to the presence of ERM; however, no statistical differences in UIVA and UNVA were observed between group 2 and the other two groups. The postoperative distant visual acuity gain was lower in eyes with primary ERM than in those of the control group in the previous study, which included all grades of ERM^21^. Postoperative visual acuity in the previous study was 0.34 logMAR, and the UDVA in our study was better than that of the previous study^21^. In terms of near vision, there was no significant difference in UNVA among the three groups; however, the proportion of patients who never or rarely need to wear near glasses was relatively lower in the group 2. Although there was no progression of ERM or increase in CST at 1 month after surgery in all groups, it has recently been reported that ERM progression after cataract surgery is associated with an abnormal vitreoretinal interface rather than presence of preoperative ERM, suggesting that the differences in CST changes between groups will be small^22^.

When measuring the axial length using partial coherence interferometry, it has been reported that an error in measurement occurs because of the change in reflectivity in presence of ERM^23,24^. However, another study reported that both partial coherence interferometry and swept source OCT-based biometer showed relatively accurate axial length measurements^25^. In our study using another swept source OCT-based biometer, the average spherical equivalent at 1 month after surgery was within -0.5 D in all groups, showing accurate IOL power calculation results despite the existence of a varying degree of ERM. A previous study reported that refractive change following 23-gauge pars plana vitrectomy for ERM in pseudophakic eyes was − 0.26 D^26^. In addition, monofocal IOL with enhanced intermediate function has the advantage of being more tolerant of residual refractive errors, which could relieve the surgeon of the risk of refractive errors following ERM surgery that may occur in the future^16,27,28^.

The group 2 showed a decreased contrast sensitivity at all spatial frequency than the other groups, but there was no statistically significant difference. Previous studies have reported that eyes with ERM have significantly reduced contrast sensitivity in all spatial frequencies than normal eyes, and Eyhance IOL shows contrast sensitivity equivalent to that of monofocal IOLs, in contrast to multifocal IOLs^16,29^. Therefore, reduced contrast sensitivity in the group 2 could be partly due to ERM itself. Because the Eyhance IOL does not reduce contrast sensitivity, it can also be useful in eyes with other ocular diseases that can reduce contrast sensitivity, such as significant vitreous floaters^30^. However, contrast sensitivity after Eyhance IOL implantation in retinal disease should be further investigated as elongation of light focusing and decreased retinal function may have a synergistic effect in eyes with retinal abnormalities. Vitrectomy with ERM peeling may result in an improvement in contrast sensitivity and visual quality in patients who experience reduced contrast sensitivity and decreased visual quality because of ERM^31^.

Regarding subjective photic phenomena, the proportion of patients who always or often experienced discomfort in the group 2 was smaller than that in the group 1 for both glare and halo, and smaller than that in the control group for glare. These results suggest that the increased discomfort following cataract surgery may be small because of poor baseline retinal image quality in the group 2. Overall satisfaction and recommendation to family or relatives were better in the group 1 and worse in the group 2 compared to the control group. There may be limitations in satisfaction in the group 2 because of the decrease in CDVA and contrast sensitivity by ERM. Nevertheless, it is considered encouraging that more than 70% of patients were satisfied after cataract surgery and willing to recommend it to others. On the other hand, despite the high occurrence of photic phenomena, the higher satisfaction of the group 1 than that of the control group is thought to be caused by the non-inferior visual acuity results and the high degree of spectacle independence.

This study is limited due to the retrospective character of the study, limited amount of patients, and short follow-up period. Long-term follow-up study with many cases is needed to support our conclusion. Moreover, there was a significant preoperative difference in CDVA between group 2 and the control group because of the presence of ERM in group 2, which limits the conclusion of this study. Since there was no comparison with controls who used a different IOLs in patients with ERM, it is insufficient to draw a conclusion that the Eyhance IOL is more useful than other IOLs. Meaningful results could be derived by a comparative study of clinical findings, including intermediate visual acuity, between monofocal IOL and Eyhance IOL implantation for patients with fovea-involving ERM.

In conclusion, patients with fovea-involving ERM showed relatively good far and intermediate visual outcomes without significant discomforts or complications, and patients with non-fovea-involving ERM showed visual outcomes comparable to those of the control group and the highest overall satisfaction after implantation of Eyhance IOL. Although a long-term follow-up study is necessary, Eyhance IOL may be a good option for patients with ERM who want better intermediate vision, regardless of whether ERM involves the fovea.

## Methods

This retrospective study reviewed the medical records of patients who underwent uncomplicated phacoemulsification with implantation of monofocal IOL with enhanced intermediate function (Eyhance IOL). Surgery was performed by one surgeon at the Department of Ophthalmology, Asan Medical Center. This study was reviewed and approved by the Institutional Review Board of the Asan Medical Center and the University of Ulsan College of Medicine (IRB No. 2022-0433) and conducted in accordance with the tenets of the Declaration of Helsinki. Signed informed consent was obtained from all participants.

The inclusion criteria were patients with preexisting ERM diagnosed by spectral-domain optical coherence tomography (SD-OCT, Heidelberg Engineering, Heidelberg, Germany) and who underwent cataract surgery with implantation of Eyhance IOL. For patients with both eyes affected, only the right eye was included for statistical analysis. The exclusion criteria were as follows: (1) patients who wanted surgical removal because of metamorphopsia and reduced contrast sensitivity due to ERM; (2) previous ocular trauma or ocular surgery, including corneal or refractive surgery; (3) corneal irregularities or abnormalities, opacities, or pathology; (4) glaucoma; (5) macular diseases, including age-related macular degeneration, diabetic macular edema, and retinal vascular occlusions; (6) use of systemic or ocular medications that affect vision and any ocular diseases other than cataract; (7) amblyopia; (8) complication during the cataract surgery; and (9) combined surgery with vitrectomy and ERM peeling.

All patients received a complete preoperative and postoperative ophthalmologic examination 1 month after surgery. All visual acuities were measured by Snellen chart and then converted to logMAR format for analysis. The preoperative examinations included uncorrected distant visual acuity (UDVA), corrected distant visual acuity (CDVA), slit-lamp examination, auto-refraction and auto-keratometry (Canon R-50, Canon USA Inc., Huntington, NY, USA), corneal topography (Orbscan, Bausch & Lomb, Rochester, NY, USA), axial length (IOL Master 700, Carl Zeiss Meditec, Jena, Germany), and central subfield thickness (CST) by SD-OCT. Cataract surgery was performed under topical anesthesia using a 2.75-mm corneal incision, manual capsulorhexis with a 6.0-mm diameter, and IOL implantation in the bag. The postoperative examination included UDVA, CDVA, uncorrected intermediate visual acuity (UIVA) at 66 cm, uncorrected near visual acuity (UNVA) at 33 cm, auto-refraction and auto-keratometry, and CST. Monocular defocus curves were measured under the photopic condition by intervals of 0.50 spherical diopters (D) from + 0.50 D to − 4.00 D.

Contrast sensitivity was measured monocularly under uncorrected conditions using the Functional Acuity Contrast Test of the Ophtec 6500 test system (Stereo Optical Co, Inc., Chicago, IL, USA) with spatial frequency at 1.5, 3, 6, 12, and 18 cycles per degree (cpd) in the photopic (85 cd/m^2^) and mesopic conditions (3 cd/m^2^).

At 1 month after surgery, patients were asked to complete a questionnaire to evaluate overall satisfaction, the occurrence of photopic visual symptoms such as glare and halo, and dependence on spectacles for near vision. Overall satisfaction was rated on a 5-point Likert scale from 1 = very dissatisfied, 2 = dissatisfied, 3 = neither satisfied nor dissatisfied, 4 = satisfied, and 5 = very satisfied. Photopic visual symptoms such as glare and halo were scored on a 5-point scale from 1 = not at all, 2 = rarely, 3 = occasionally, 4 = often, and 5 = always. Patients were surveyed for the recommendation of Eyhance IOL implantation to other people, with allowed responses being yes or no.

### ERM classification

**Figure 6:**
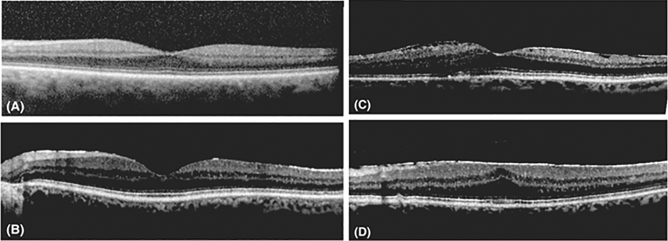
Epiretinal membrane (ERM) staging by spectral-domain optical coherence tomography. (**A**) stage 0: absence of a continuous hyperreflective signal at the inner retinal surface, (**B**) stage 1 or continuous hyperreflectivity: the presence of a continuous hyperreflective signal at the inner retinal surface on at least three consecutive sections of the macular cube (to limit confusion with posterior hyaloid reflectivity), (**C**) stage 2 or mature ERM without foveal involvement: stage 1 associated with retinal folds but without alterations of the foveal depression, and (**D**) stage 3 or mature ERM with foveal involvement: stage 2 associated with foveal depression alterations.

SD-OCT was performed before and 1 month after surgery. The regional retinal thickness according to the 1-mm, 3-mm, and 6-mm early treatment diabetic retinopathy Study (ETDRS) map was obtained by the built-in program on the SD-OCT instrument. The analyses of SD-OCT were done to analyze regional retinal thickness and the severity of ERM. The stage of the ERM was defined as follows on SD-OCT scans, as previously done by Delyfer et al.: stage 0: absence of a continuous hyperreflective signal at the inner retinal surface (Fig. 6A), stage 1 or continuous hyperreflectivity: the presence of a continuous hyperreflective signal at the inner retinal surface on at least three consecutive sections of the macular cube (to limit confusion with posterior hyaloid reflectivity) (Fig. 6B), stage 2 or mature ERM without foveal involvement: stage 1 associated with retinal folds but without alterations of the foveal depression (Fig. 6C), and stage 3 or mature ERM with foveal involvement: stage 2 associated with foveal depression alterations (Fig. 6D)^32^. We divided the patients into three groups according to ERM grading. Group 1 was a non-fovea-involving ERM group, defined as stage 1 to stage 2 without fovea involvement. Group 2 was a fovea-involving ERM group, defined as stage 3 with fovea involvement and alteration of the foveal depression. A control group was selected from patients without ERM and underwent surgery during the study period through age matching method.

### Statistical analysis

Statistical analysis was done using the Statistical Package for the Social Sciences software for Windows version 21.0 (IBM, SPSS Inc., Chicago, IL, USA). A Wilk-Shapiro test was used to assess the distribution of numerical data. Preoperative data and postoperative outcomes were compared for each eye using the Wilcoxon signed-rank test. The mean value for visual outcomes, defocus curves, patients’ satisfaction, and spectacle independence were compared between groups using the Kruskal–Wallis test with Bonferroni post-hoc correction. *P*-values less than 0.05 were considered statistically significant.

## Data Availability

The datasets generated during and/or analyzed during the current study are available from the corresponding author on reasonable request.
